# Correction to: Deciphering cellular transcriptional alterations in Alzheimer’s disease brains

**DOI:** 10.1186/s13024-020-00403-6

**Published:** 2020-09-15

**Authors:** Xue Wang, Mariet Allen, Shaoyu Li, Zachary S. Quicksall, Tulsi A. Patel, Troy P. Carnwath, Joseph S. Reddy, Minerva M. Carrasquillo, Sarah J. Lincoln, Thuy T. Nguyen, Kimberly G. Malphrus, Dennis W. Dickson, Julia E. Crook, Yan W. Asmann, Nilüfer Ertekin-Taner

**Affiliations:** 1 Department of Health Sciences Research, Mayo Clinic Florida, Jacksonville, FL USA; 2 Department of Neuroscience, Mayo Clinic Florida, Jacksonville, FL USA; 3 Department of Mathematics and Statistics, University of North Carolina at Charlotte, Charlotte, NC USA; 4 Department of Neurology, Mayo Clinic Florida, Jacksonville, FL USA

**Correction to: Mol Neurodegeneration (2020) 15:38**

**https://doi.org/10.1186/s13024-020-00392-6**

After publication of this article [1], the author reported that Figure S8 in Additional File 1 contains tracks of insertion, which should have been accepted. The updated Additional File 1 is shown in the Additional File section below.

## Supplementary information

**Additional file 1.**
